# Dowstream events in the NIK-mediated defense associated with resistance to begomovirus

**DOI:** 10.1186/1753-6561-8-S4-O22

**Published:** 2014-10-01

**Authors:** Elizabeth Fontes

**Affiliations:** 1 Departamento de Bioquímica e Biologia Molecular, National Institute of Science and Technology in Plant-Pest Interactions/ Bioagro- Universidade Federal de Viçosa, Viçosa, MG, Brazil

## 

The NSP-interacting kinase (NIK) receptor-mediated antiviral signaling has been identified as a virulence target of the begomovirus nuclear shuttle protein (NSP) [1,2]. NSP suppresses the activity of the NIK receptor through specific binding to the kinase domain and hence enhances begomovirus pathogenicity [1,3]. NIK receptors belong to the plant defense group of the leucine-rich repeat (LRR) receptor-like kinase (RLK) subfamily, designated LRR-RLK II. This subfamily of RLKs from tomato and Arabidopsis is constituted by 14 proteins harboring four complete LRRs (with 24 residues) and a fifth incomplete LRR (with 16 residues) arranged in a single continuous block within the extracellular domain [4]. Based on sequence conservation and structural features, the members of the LRR-RLK II subfamily are clustered into three distinct branches: (i) antiviral defense proteins (ii) developmental and defense proteins, such as the somatic embryogenesis receptor-like kinases (SERK-like) including SERK1 and SERK3/BAK1 and (iii) functionally unassigned proteins. The Arabidopsis NSP-interacting kinase 1, NIK1 (At5g16000), NIK2 (At3g25560) and NIK3 (At1g60800) are in the defense group I of the LRR-RLKII subfamily and are virulence targets of the bipartite geminivirus nuclear shuttle protein, NSP [1,4]. NSP from CaLCuV (*Cabbage leaf curl virus *interacts with all three NIKs from Arabidopsis to suppress their kinase activity [4]. The NSP-NIK interaction is also conserved among geminivirus NSPs and NIK homologs from different hosts [2]. Tomato and soybean NIK homologs also interact stably with NSP from CaLCuV [2] and with NSPs from the tomato-infecting geminiviruses TGMV (*Tomato golden mosaic virus*), TCrLYV (Tomato crinkle leaf yellows virus) and ToYSV (*Tomato yellow spot virus*) [1,2]. Several lines of evidence indicate that NIK functions in defense. NSP from CaLCuV acts as a virulence factor to suppress the kinase activity of transmembrane receptor NIKs [1]. Second, loss of NIK1, NIK2 or NIK3 function in Arabidopsis is also linked to an enhanced susceptibility phenotype to CaLCuV infection [1,3]. In addition, overexpression of NIK1 from Arabidopsis in tomato plants attenuates symptom development and delays ToYSV infection [5]. NIK exhibits trans-autophosphorylation activity in vitro and substrate phosphorylation activity *in vitro *and *in vivo*, and interacts with the ribosomal proteins L10 (rpL10) and L18 (rpL18) [5]. NIK-mediated phosphorylation of rpL10 promotes translocation of the ribosomal protein to the nucleus where it may function to mount a defense response that negatively impacts virus infection. This is consistent with the notion that the regulated nucleocytoplasmic shuttling of rpL10 links the antiviral response to receptor activation. We found that NIK1 undergoes a stepwise pattern of phosphorylation within its activation-loop domain (A-loop) with distinct roles for different threonine residues [6]. The conserved Thr-474 and Thr-469 were found to be phosphorylated in vitro and mutations at Thr-474 impaired autophosphorylation and were defective for kinase activation in vitro and in vivo. In contrast, a mutation at Thr-469 did not impact autophosphorylation and increased substrate phosphorylation, suggesting an inhibitory role for Thr-469 in kinase function. Our results establish that NIK1 functions as an authentic defense receptor as it requires activation to elicit a defense response [6,7]. Our data also suggest a model whereby phosphorylation-dependent activation of a plant receptor-like kinase enables the A-loop to control differentially auto- and substrate phosphorylation.

To identify novel regulators of NIK-mediated defense response, we screened a two-hybrid library for partners of rpL10. We discovered a novel transcriptional factor, which interacts with rpL10 in the nucleus of plant cells to down-regulate the expression of ribosomal genes. These data are consistent with the observation that constitutive activation of the NIK receptor by replacing Thr-474 with aspartate impairs global translation in Arabidopsis and tomato transgenic lines and confers broad-spectrum tolerance to begomovirus infection. Our data also indicate that the NIK immune receptor-mediated antiviral signaling operates through a bipartite module. One defense signal-transducing branch is mediated by the regulated nucleocytoplasmic transport of ribosomal protein to impair translation and the second branch of the antiviral signaling transduces a typical defense signal through induction of the plant immune system. We will discuss novel insights into the regulation of the NIK-mediated antiviral signaling and an efficient-acquired defense against begomovirus by modulating the activity of the immune defense receptor NIK in tomato plants.
